# Is Kaposi’s sarcoma the end of the OX40/OX40L axis in atopic dermatitis?

**DOI:** 10.3389/fimmu.2026.1870289

**Published:** 2026-06-02

**Authors:** Shahram Salek-Ardakani

**Affiliations:** YZ Consulting, La Jolla, CA, United States

**Keywords:** antiviral immunity, atopic dermatitis, drug safety, HHV-8, inflammation, Kaposi’s sarcoma, OX40 (TNFRSF4), OX40L (TNFSF4)

## Abstract

The OX40/OX40L axis entered clinical development in atopic dermatitis with a strong biological rationale and early signs of durable activity. However, as the treatment landscape evolved, questions emerged about whether the magnitude of monotherapy benefit was sufficient relative to established and emerging therapies. The discontinuation of rocatinlimab after confirmed and suspected cutaneous Kaposi’s sarcoma cases, together with two cumulative cases reported in the amlitelimab program in patients with known risk factors, has changed the discussion from early promise to mechanism, risk, and therapeutic strategy. Although a causal link between OX40/OX40L modulation and Kaposi’s sarcoma remains unproven, available human genetic and experimental observations make the association biologically plausible but mechanistically unresolved. The central challenge is now to determine how the axis can be targeted, in which patients, and in what therapeutic context, to maximize clinical benefit while managing risk. Rather than signaling the end of the axis in atopic dermatitis, Kaposi’s sarcoma may instead mark the limits of a first-generation development strategy and the beginning of a more selective approach built around molecule design, therapeutic context, and prospective risk mitigation.

## Introduction

Interest in the OX40/OX40L axis in atopic dermatitis (AD) stemmed from its promise to deliver deeper and more durable effects than conventional cytokine blockade. However, recent cases of Kaposi’s sarcoma in two leading programs—rocatinlimab, an anti-OX40 antibody originally developed by Kyowa Kirin and later advanced with Amgen as KHK4083/AMG 451, and amlitelimab, Sanofi’s anti-OX40L antibody SAR445229/KY1005—have made that promise more difficult to interpret and raised the standard for future development. The field must now assess whether OX40/OX40L targeting can provide meaningful clinical benefit without introducing significant vulnerabilities in antiviral immune surveillance.

Atopic dermatitis is a chronic, heterogeneous inflammatory disease characterized by recurrent lesions, pruritus, pain, sleep disturbance, and substantial quality-of-life burden (1–3). Its pathogenesis reflects the interaction of environmental triggers, epithelial barrier dysfunction, microbial dysbiosis, and immune dysregulation, with T cell-mediated inflammation playing a central role (3, 4). The success of dupilumab, an IL-4Rα antibody that inhibits IL-4 and IL-13 signaling, together with Janus kinase (JAK) inhibitors, has transformed the treatment of moderate-to-severe disease and established targeted immune modulation as a highly effective strategy (1, 2). Still, many patients do not achieve adequate disease control, and some discontinue treatment because of adverse effects, loss of efficacy, incomplete response, or treatment burden (1, 2). These unmet needs leave room for new mechanisms, but the success of existing therapies means that incremental efficacy alone is no longer enough. New biologics must now offer a clearer rationale for use, whether through greater depth or durability of response, greater convenience, improved safety, differentiated patient selection, or a credible role in sequencing and combination therapy.

The biological rationale for OX40/OX40L in AD was strong. OX40 is induced on activated T cells, whereas OX40L is upregulated on antigen-presenting cells and structural cells in inflamed skin (5, 7–11). In AD, both are increased in lesional tissue, correlate with disease activity, and decline with effective treatment (5, 7–11). Functionally, the pathway has been linked to the survival and reactivation of pathogenic CD4 and CD8 T cells, the maintenance of immune memory, and persistent tissue crosstalk (5, 6). Collectively, these features suggested that the axis might influence not only acute inflammation but also the cellular and molecular programs that contribute to chronicity and recurrent flares.

**Figure 1:**
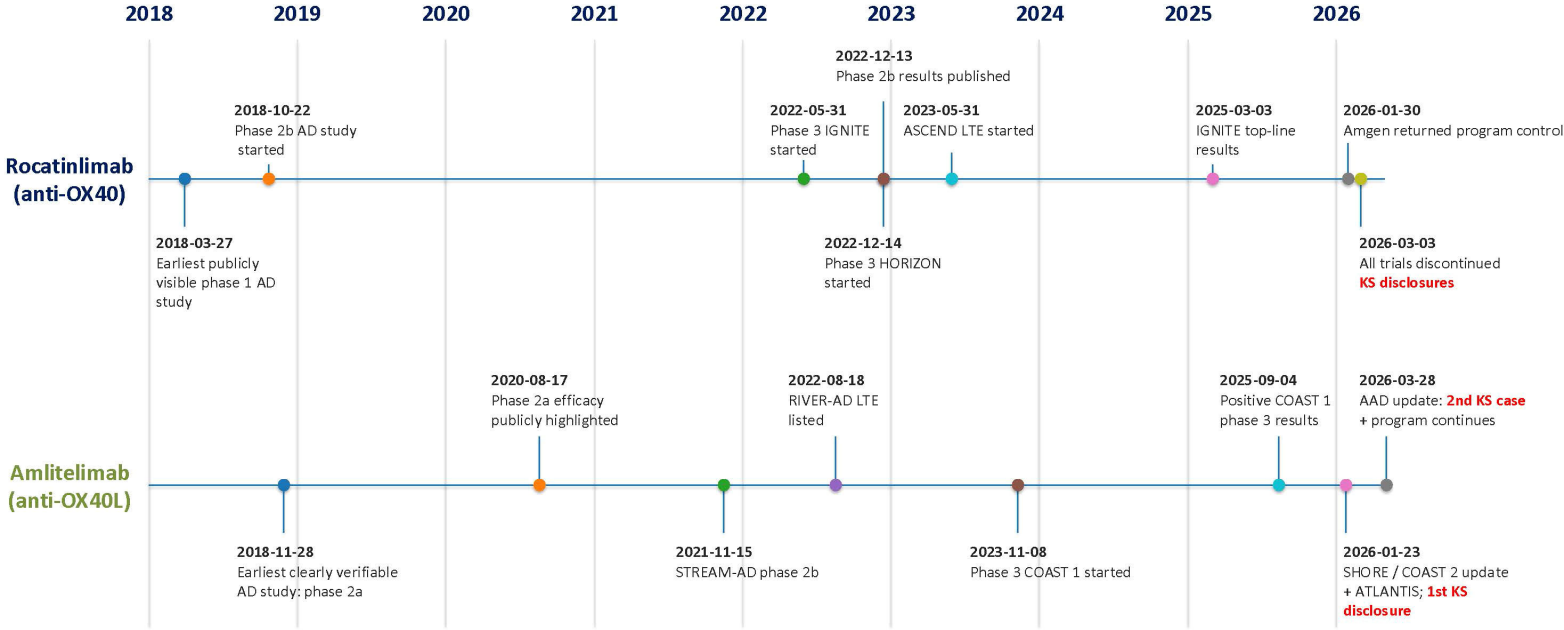
Development timeline of OX40- and OX40L-directed programs in atopic dermatitis. The clinical development trajectories of the two leading programs targeting the OX40/OX40L axis in atopic dermatitis: rocatinlimab (KHK4083/AMG 451), an anti-OX40 antibody, and amlitelimab (KY1005/SAR445229), an anti-OX40L antibody. The timeline highlights early clinical entry, proof-of-concept studies, phase 2 and phase 3 milestones, longer-term follow-up studies, major efficacy readouts, and the key safety and strategic inflection points that reshaped the field in 2026. ClinicalTrials.gov identifiers shown where available: KHK4083 phase 1, NCT03096223; rocatinlimab phase 2b, NCT03703102; ROCKET-IGNITE, NCT05398445; ROCKET-HORIZON, NCT05651711; ROCKET-SHUTTLE, NCT05724199; ROCKET-ASTRO, NCT05704738; ROCKET-ORBIT, NCT05633355; ROCKET-VOYAGER, NCT05899816; ROCKET-ASCEND, NCT05882877; amlitelimab phase 2a, NCT03754309; STREAM-AD, NCT05131477; ATLANTIS, NCT05769777; COAST 1, NCT06130566; COAST 2, NCT06181435; SHORE, NCT06224348; AQUA, NCT06241118; ESTUARY, NCT06407934.

Clinical data initially reinforced this rationale (**Figure 1**) (12–17). Early- and mid-stage studies of rocatinlimab and amlitelimab showed that targeting either the receptor or the ligand could produce meaningful clinical activity in AD (12–17). Notably, responses to rocatinlimab persisted through week 36 and, in some patients, beyond treatment discontinuation (12–14). Amlitelimab similarly suggested that OX40L blockade could produce clinical improvement that may deepen over time or persist after treatment withdrawal in some settings (15–18, 21, 24). Together, these observations helped define OX40/OX40L as a clinically active axis. However, they did not resolve the harder question of whether this activity was sufficiently differentiated to support broad monotherapy development in an increasingly competitive AD field.

This unresolved positioning became more urgent in early 2026, as safety concerns moved from theoretical to program-defining. On January 23, 2026, Sanofi reported a case of cutaneous Kaposi’s sarcoma in the ATLANTIS open-label extension, noting that the patient had known risk factors (18). One week later, Kyowa Kirin announced Amgen would return the rocatinlimab program, attributing the decision to portfolio strategy rather than new safety concerns (19). The more consequential development came on March 3, 2026, when Kyowa Kirin halted all rocatinlimab trials after a safety review identified two confirmed cases of Kaposi’s sarcoma and one suspected case, and concluded that a link to modulation of the OX40 pathway could not be excluded (20). Finally, on March 28, 2026, Sanofi added further uncertainty by reporting a second case of Kaposi’s sarcoma in the still-blinded ESTUARY phase 3 study—again, in a patient with known risk factors (21).

Collectively, these events changed the question facing OX40/OX40L development in AD. Kaposi’s sarcoma, a human herpesvirus 8 (HHV-8)-associated malignancy, is not simply an adverse event to be monitored. Rather, it introduces a safety question that must be incorporated into future development without negating the rationale for targeting OX40/OX40L in AD. If OX40/OX40L contributes to both pathogenic T-cell persistence and functionality in AD and immune control of HHV-8 in susceptible individuals, the main challenge is to determine whether the anti-inflammatory benefits of pathway modulation can be separated from immune-surveillance risks and whether those risks can be prospectively identified and mitigated.

## Clinical activity is established, but therapeutic positioning remains unclear

Clinical experience with rocatinlimab and amlitelimab has clarified both the therapeutic promise and the limitations of OX40/OX40L targeting in AD. Both programs produced efficacy signals consistent with pharmacological engagement of the pathway. However, neither program fully defines where OX40/OX40L-directed therapy fits in an AD landscape now shaped by effective biologics, emerging agents, and rising expectations for safety, convenience, and depth of response.

Rocatinlimab showed that OX40 targeting could translate into meaningful clinical benefit in AD (12–14). In phase 2b, the most active regimens produced adjusted mean EASI reductions of 50–61% at week 16, compared with 15% for placebo (13). This was then further confirmed in phase 3 (22, 23). In HORIZON monotherapy, week-24 vIGA-AD 0/1 and EASI-75 rates were 19.3% and 32.8%, compared with 6.6% and 13.7% for placebo, with improvement also observed in itch, pain, quality of life, and hand and facial dermatitis (22, 23). IGNITE extended these data into a more treatment-experienced population, including patients previously exposed to biologics or JAK inhibitors (22, 23). At week 24, EASI-75 was achieved in 42.3% and 36.3% of the two active-dose groups, with placebo-adjusted differences of 29.5% and 23.4%, while vIGA-AD 0/1 rates were 23.6% and 19.1%, corresponding to placebo-adjusted differences of 14.9% and 10.3% (22, 23). Longer-term follow-up from ASCEND suggested that the benefit could be maintained with less frequent dosing, supporting the idea that OX40 targeting may have effects beyond short-term inflammatory suppression (22, 23). These results provided important validation for receptor-directed targeting, but they also illustrate the narrow margin facing new AD biologics. Once a serious, mechanistically plausible safety signal emerged, the same efficacy profile had to justify a much higher burden of safety monitoring, risk mitigation, and development complexity.

Amlitelimab raises a related question for ligand blockade. In phase 2a, one dose group achieved an adjusted mean EASI reduction of about 80% at week 16, compared with 49% for placebo (15, 16), and phase 2b similarly showed improvement while suggesting that some benefit could persist after treatment withdrawal (16, 24). The phase 3 program maintained that overall efficacy signal but made the picture more complex by showing greater variability across trial settings (18, 21). In SHORE, which included background topical therapy, week-24 vIGA-AD 0/1 rates were 28.7% and 32.3%, compared with 16.8% for placebo, while EASI-75 rates were 48.1% and 46.8%, compared with 32.3% for placebo (18, 21). COAST 1 met key endpoints, whereas COAST 2 showed mixed results, with US analyses favoring amlitelimab but EU analyses failing to show a statistically significant benefit in the Q12W cohort under the prespecified testing hierarchy (18, 21). Open-label follow-up in ATLANTIS added a longer-term perspective, with vIGA-AD 0/1 increasing from 35.4% at week 24 to 50.3% at week 52, while EASI-75 rose from 62.9% to 76.5%; however, the absence of a concurrent control group limits interpretation of apparent response deepening over time (18, 21). Together, these findings support OX40L blockade as a credible approach, particularly if gradual deepening of response and less frequent dosing prove clinically meaningful.

Across both programs, the data show that OX40/OX40L can be therapeutically engaged, but they do not yet define the threshold for continued development. The potential for progressive or durable benefit remains significant, particularly in a disease characterized by chronicity and relapse, but it must be weighed against the modest-to-moderate efficacy observed in some monotherapy settings, the competitive therapeutic landscape, and the complexity introduced by a serious safety signal. The central question is therefore no longer whether OX40/OX40L is active, but whether its clinical impact is sufficiently robust and manageable to warrant continued development in its current form. Addressing this issue will require identifying clinical contexts in which OX40/OX40L biology is most relevant and where the observed benefits justify continued development.

## Kaposi’s sarcoma links OX40/OX40L targeting to immune-surveillance risk

The Kaposi’s sarcoma cases changed the OX40/OX40L discussion by linking therapeutic modulation of chronic T-cell-driven inflammation to a possible vulnerability in antiviral host defense. This concern is most relevant in patients with latent HHV-8 infection or other predisposing risk factors, where the same pathway being targeted in AD may also contribute to viral control. The key question is whether these cases reflect risk concentrated in susceptible patients, molecule-specific effects, or a broader pathway-related liability.

At present, the clinical importance of this risk is difficult to quantify because many case-level details remain undisclosed, including baseline risk factors, HHV-8 status, geographic background, duration of exposure, concomitant immunomodulation, and timing relative to treatment response. What is publicly known is that two cumulative cases of Kaposi’s sarcoma have been reported, both in patients with known risk factors, with one occurring in the ATLANTIS open-label extension and the other in the still-blinded ESTUARY phase 3 study (18, 21). In Sanofi’s March 2026 update, these two cases occurred among 3,778 patients confirmed to have been exposed to amlitelimab across indications, corresponding to an apparent reported frequency of approximately 0.05%. No additional cases were identified across an estimated 4,630 patients in the full amlitelimab development program, including blinded studies (18–21). Thus, the reported frequency appears low, but the cases remain clinically important because the specific risk profile of affected patients has not been fully defined. For rocatinlimab, even less has been disclosed. Kyowa Kirin stated that a mechanistic link to OX40 modulation could not be excluded but released limited clinical detail before terminating the program (20). Overall, the signal is important enough to reshape benefit-risk assessment, but the available data do not yet define its incidence, mechanism, or distribution across patient subgroups. At present, these cases should be viewed as a signal requiring mechanistic and clinical clarification rather than as evidence of a class-wide effect.

Kaposi’s sarcoma is a human herpesvirus 8 (HHV-8)-associated endothelial neoplasm that emerges in the setting of impaired host immune surveillance (25). This gives the clinical cases a credible biological context, even though causality remains unresolved. The most direct evidence linking OX40 biology to Kaposi’s sarcoma comes from human genetics. Byun and colleagues described a 19-year-old Turkish woman with childhood-onset classic Kaposi’s sarcoma who carried an autosomal recessive loss-of-function mutation in OX40 (26). Their findings placed OX40 deficiency within a larger group of inborn errors of immunity associated with childhood Kaposi’s sarcoma, alongside defects in IFN-γR1, Wiskott–Aldrich syndrome, and stromal interaction molecule 1 (STIM1) (25, 26). They further proposed that OX40 contributes to immune control of HHV-8 and may be particularly important for memory CD4 T-cell function in this setting (26). The observation of abundant OX40L expression in AIDS-related Kaposi’s sarcoma lesions was consistent with this argument, suggesting that the axis may be relevant to both systemic antiviral control and local protection against HHV-8-driven endothelial transformation (26). These observations support a link between OX40 biology and Kaposi’s sarcoma, but they do not establish that therapeutic OX40 or OX40L modulation reproduces congenital OX40 deficiency. This limitation is important because congenital OX40 deficiency represents lifelong, complete loss of function, whereas therapeutic modulation is partial, time-limited, and pharmacologically distinct.

The details of the OX40-deficiency case also argue against a simple model of broad immune suppression. The patient’s phenotype was consistent with defective CD4 memory function, including reduced circulating effector-memory CD4 T cells and impaired recall responses to several antigens. However, HHV-8-specific CD4 or CD8 T-cell recall responses were not directly assessed, making it difficult to definitively link the findings to impaired HHV-8 control (26). Notably, the broader immune profile did not suggest generalized dysfunction. Total leukocyte counts were normal, humoral immunity was largely intact, and no overt abnormalities were detected across a broad panel of T-cell differentiation and effector markers (26). Although CD8 effector-memory T cells were somewhat reduced, circulating CMV- and EBV-specific CD8 T cells remained detectable (26). The more subtle interpretation is that OX40 deficiency may reveal a selective defect in antiviral memory or recall responses, one that could be missed by conventional immune monitoring and become clinically relevant only in the right viral, genetic, geographic, or immunological context. The same congenital OX40-deficiency case also included visceral leishmaniasis, suggesting that susceptibility may not be limited to HHV-8 or herpesvirus immunity (26). More broadly, OX40 biology could plausibly influence immune control of selected persistent pathogens or tumor surveillance, although public clinical disclosures for rocatinlimab and amlitelimab have not established a broader pattern of persistent infection or malignancy. At present, Kaposi’s sarcoma remains the primary clinically recognized signal, while broader pathogen- and tumor-surveillance risks remain theoretical and should be monitored prospectively.

Experimental data are limited but directionally consistent with this interpretation. Cho and Myoung reported that recombinant OX40 suppressed HHV-8 lytic replication in lymphatic endothelial cells, suggesting that intact OX40–OX40L interactions may help restrain viral replication in a cellular context relevant to Kaposi’s sarcoma pathogenesis (27). However, the study was performed *in vitro*, the effect was not seen across all relevant cell types, and the role of OX40L reverse signaling in endothelial cells was not clearly defined (27). The experimental evidence therefore adds to the rationale, but it remains too limited to establish a firm mechanistic link between therapeutic pathway modulation and the Kaposi’s sarcoma cases reported in clinical development.

Taken together, the Kaposi’s sarcoma signal should be treated as serious and biologically plausible, but not yet resolved. The currently reported frequency appears low, at least for amlitelimab, but the clinical significance of even rare events depends on whether risk is predictable, preventable, and concentrated in identifiable patient groups. The current evidence therefore does not warrant dismissing the risk, nor does it establish class-wide liability. Future work should determine whether susceptibility varies by molecular format, particularly between receptor-directed depletion and ligand blockade, and whether it is concentrated among patients with HHV-8 infection, relevant epidemiological exposure, or other predisposing factors. It should also assess whether this risk can be mitigated through patient selection, exclusion criteria, monitoring, or alternative drug designs. These considerations define the mechanistic uncertainty now surrounding the axis and should set the parameters for future development.

## The future of OX40/OX40L depends on improving benefit-risk

The future of the OX40/OX40L axis in atopic dermatitis may depend as much on how the pathway is targeted as on whether it remains therapeutically useful. The recent Kaposi’s sarcoma signal has highlighted the limitations of treating rocatinlimab and amlitelimab as interchangeable approaches (18–21). Although both agents engage the same pathway, they do so through different mechanisms. Rocatinlimab targets OX40 on activated T cells and appears to deplete these cells, whereas amlitelimab blocks OX40L without depleting OX40- or OX40L-expressing cells (5, 6). This distinction is particularly relevant if OX40 biology contributes to antiviral immunity. In this context, receptor-directed depletion may exert broader and more durable effects on activated T-cell populations, possibly increasing vulnerability to latent or opportunistic viral infections, whereas non-depleting ligand blockade may interrupt signaling while preserving a greater proportion of immune cells necessary for surveillance. This does not establish that OX40L blockade is safer than OX40 targeting, but it makes the distinction between depletion and non-depletion a central question for future development. The more meaningful comparison, therefore, may not be between stronger and weaker versions of the same mechanism, but between approaches that perturb the pathway in materially different ways, with potentially different implications for efficacy, safety, patient selection, and monitoring.

The most compelling role for OX40/OX40L may be in modifying disease persistence and durability of response, rather than rapidly suppressing early disease activity. Such effects may not be fully captured by monotherapy endpoints focused on early clinical improvement. The challenge, therefore, is to integrate OX40/OX40L targeting into treatment strategies that make use of these strengths. As monotherapy, modest-to-moderate efficacy may not be sufficient to justify the added complexity of screening, excluding, and monitoring patients for rare but serious immune-surveillance risks. One possible approach is to evaluate OX40/OX40L targeting in combination therapy, rational sequencing, or multifunctional formats, particularly with mechanisms that address pathogenic innate and adaptive immune programs, barrier dysfunction, or pruritus (1). In principle, combining OX40/OX40L modulation with orthogonal inflammatory mechanisms could improve depth of response, but this would need to be demonstrated without introducing disproportionate infection, malignancy, or immune-surveillance risk. In this setting, the additional operational burden of Kaposi’s sarcoma risk mitigation would need to be justified by a clearly greater clinical benefit. If successful, such strategies could allow OX40/OX40L modulation to support more durable disease control, while complementary mechanisms provide faster or deeper suppression of active inflammation.

This shift in therapeutic positioning is already evident in how the pathway is being developed. Sanofi continues to advance amlitelimab as a non-depleting OX40L-directed therapy in AD, even as recent clinical and safety developments have made the case for stand-alone monotherapy more complex. Updated phase 3 results remain consistent with support for Q12W dosing from the start, while longer-term observations suggesting gradual response deepening should be interpreted cautiously where they derive from uncontrolled extension data (18, 21). The same logic is reflected in broader multifunctional designs, including brivekimig, an OX40L x TNF Nanobody^®^ (28), as well as publicly disclosed OX40L x IL-13 and other next-generation bispecific approaches. Although these programs remain early, they suggest that future progress may depend less on OX40L blockade alone than on combining pathway modulation with other relevant elements of AD biology. The opportunity is not simply to change molecular format, but to improve the therapeutic index. This will require stronger efficacy, clearer patient selection, prospective screening for Kaposi’s sarcoma susceptibility, and monitoring strategies that make rare but serious risks manageable. The experience with JAK inhibitors is instructive. Boxed warnings and serious safety concerns have not eliminated their use in AD, but they have shaped patient selection, labeling, monitoring, and the clinical contexts in which benefit is judged to outweigh risk. The strategic opportunity, therefore, may be less about establishing that OX40/OX40L can stand alone and more about identifying the settings in which its effects on persistence and durability add value to complementary mechanisms without imposing an unacceptable safety burden.

## Discussion

Kaposi’s sarcoma has changed the development context for the OX40/OX40L axis in atopic dermatitis. The clinical data from rocatinlimab and amlitelimab establish that the pathway can be therapeutically engaged. The unresolved question is whether OX40/OX40L modulation can deliver sufficient clinical benefit in the right patients and with the right molecular format to justify the safety and development complexity now attached to the axis. This issue became more urgent after rocatinlimab’s discontinuation and more complex as the amlitelimab dataset brought both progressive efficacy and unresolved safety questions into sharper focus (18, 21).

The immediate priority is to define risk in a mechanistic, clinically actionable, and prospectively testable manner. Future studies should determine whether OX40/OX40L modulation is associated with increased risk of Kaposi’s sarcoma, which components of antiviral immune surveillance are affected, and whether liability differs between receptor-directed depletion and ligand blockade. They should also define whether susceptibility is enriched in specific patient populations, including those with HHV-8 infection, relevant geographic or epidemiological exposure, prior immunosuppression, HIV infection where relevant, transplant or malignancy history, or markers of impaired antiviral immunity. Future trials should therefore integrate baseline virological and immunological risk profiling, geography-aware enrollment, predefined high-risk subgroups, robust monitoring, and long-term follow-up for delayed adverse events, including rare malignancies. These designs should distinguish pathway-related, molecule-specific, and host-context-dependent risk while testing whether patient selection, exclusion criteria, enhanced monitoring, or alternative molecular designs can reduce it. For amlitelimab specifically, ESTUARY will be an important test of whether the signals of progressive efficacy, Q12W maintenance, and longer-term safety can be more clearly aligned. Combination therapy, sequencing, and multifunctional design remain important, but their value will depend on whether they increase clinical benefit enough to justify any added safety or monitoring burden.

At present, the most appropriate conclusion is neither reassurance nor abandonment. The OX40/OX40L axis remains both biologically and clinically credible in AD, but future development will require a more precise approach to molecular format, patient susceptibility, and therapeutic context. The available evidence supports continued evaluation of whether risk is concentrated in identifiable populations, whether it can be mitigated prospectively, and whether clinical benefit is sufficient to justify additional monitoring and development complexity. In this context, the recent Kaposi’s sarcoma signal may represent not the end of the axis, but a transition toward a more precise and biologically informed phase of development.

## Data Availability

The original contributions presented in the study are included in the article/supplementary material. Further inquiries can be directed to the corresponding author.
